# Quantitative pulmonary pharmacokinetics of tetrandrine for SARS-CoV-2 repurposing: a physiologically based pharmacokinetic modeling approach

**DOI:** 10.3389/fphar.2024.1457983

**Published:** 2024-09-13

**Authors:** Furun Wang, Liuhan Dong, Juanwen Hu, Shijie Yang, Lingchao Wang, Zhiwei Zhang, Wenpeng Zhang, Xiaomei Zhuang

**Affiliations:** ^1^ State Key Laboratory of Toxicology and Medical Countermeasures, Beijing Institute of Pharmacology and Toxicology, Beijing, China; ^2^ Huadong Medical Institute of Biotechniques, Nanjing, China

**Keywords:** tetrandrine, physiologically based pharmacokinetic (PBPK), pulmonary exposure, drug repurpose, lysosomal trapping

## Abstract

Tetrandrine (TET) has been traditionally used in China as a medication to treat silicosis and has recently demonstrated anti-SARS-CoV-2 potential *in vitro*. By recognizing the disparity between *in vitro* findings and *in vivo* performance, we aimed to estimate the free lung concentration of TET using a physiologically based pharmacokinetic (PBPK) model to link *in vitro* activity with *in vivo* efficacy. Comparative pharmacokinetic studies of TET were performed in rats and dogs to elucidate the pharmacokinetic mechanisms as well as discern interspecies variations. These insights facilitated the creation of an animal-specific PBPK model, which was subsequently translated to a human model following thorough validation. Following validation of the pharmacokinetic profile from a literature report on single oral dosing of TET in humans, the plasma and lung concentrations were predicted after TET administration at approved dosage levels. Finally, the antiviral efficacy of TET in humans was assessed from the free drug concentration in the lungs. Both *in vivo* and *in vitro* experiments thus confirmed that the systemic clearance of TET was primarily through hepatic metabolism. Additionally, the lysosomal capture of basic TET was identified as a pivotal factor in its vast distribution volume and heterogeneous tissue distribution, which could modulate the absorption dynamics of TET in the gastrointestinal tract. Notably, the PBPK-model-based unbound lung concentration of TET (1.67–1.74 μg/mL) at the recommended clinical dosage surpassed the *in vitro* threshold for anti-SARS-CoV-2 activity (EC_90_ = 1.52 μg/mL). Thus, a PBPK model was successfully developed to bridge the *in vitro* activity and *in vivo* target exposure of TET to facilitate its repurposing.

## 1 Introduction

**FIGURE 1:**
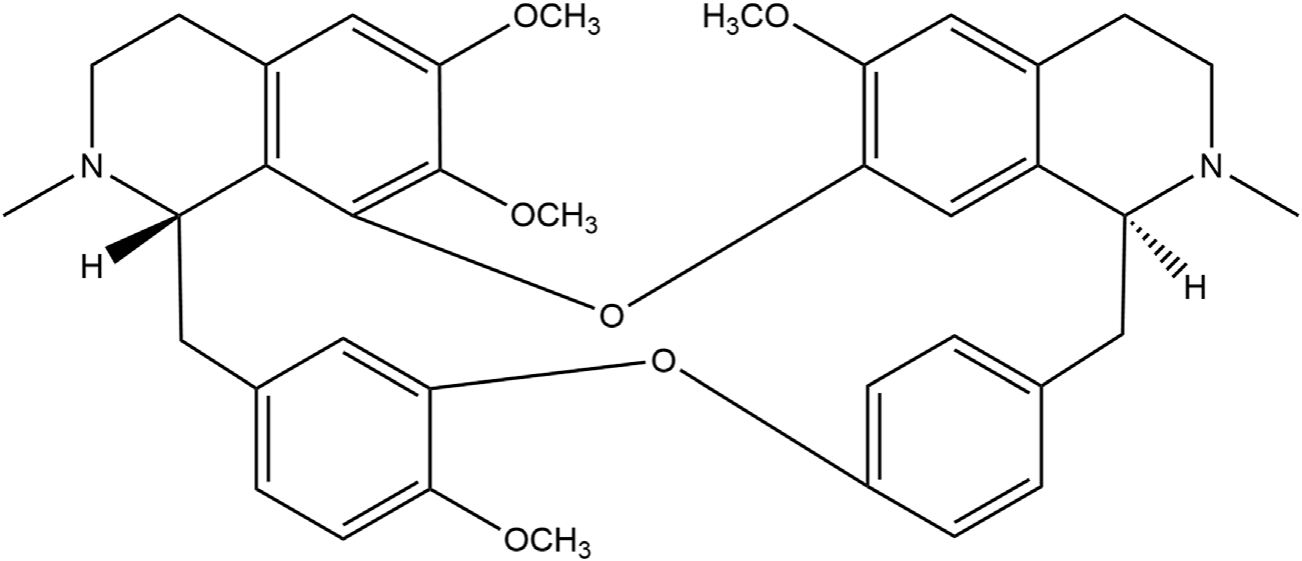
Chemical structure of tetrandrine (TET).

The recent COVID-19 pandemic has highlighted a critical need for antiviral drugs, particularly with respect to prevention of development of respiratory infections and as treatment medications. Tetrandrine (TET, Figure 1) is a benzylisoquinoline alkaloid extracted from the herb *Stephania tetrandra* S. Moore that has been utilized in China for over 50 years to treat various respiratory illnesses, including silicosis; its antagonistic effects on Ca^2+^ channels are well-documented (Liu et al., 1995; Jiang et al., 2020). Recent research suggest that TET may protect cells against viral infections by blocking the TPC2-Ca^2+^ pathway on the lysosomes, thereby hindering viral entry into host cells (Grimm and Tang, 2020; Heister and Poston, 2020; Ou et al., 2020).

Our recent studies have yielded promising results on the efficacy of TET against SARS-CoV-2 *in vitro* and its significant alleviation of murine lung injuries caused by SARS-CoV-2 infection. Pharmacokinetic studies have shown that the blood concentration of TET after intravenous (i.v.) dosing is relatively low. However, the metabolic clearance of TET in animals is not high and shows a large distribution volume (V) primarily in lysosome-rich tissues, including the lungs, liver, spleen, kidneys, and intestines; it was observed that the area under the curve (AUC) for the lung could be over 25 times higher than that in whole blood (Liu et al., 2023).The inflammatory activation of lysosomal trapping and inhibition of metabolic enzymes can lead to significant increases in lung concentrations (AUCs can be about 50 times higher than that in whole blood) (Wang et al., 2022). However, efficiently and reasonably translating *in vitro* pharmacological activity to real-world efficacy is a challenge, particularly in the context of repurposing approved medicines (Saeidnia et al., 2015; Daoud et al., 2021).

Although TET has demonstrated efficacy in the clinical treatment of silicosis, human pharmacokinetic data remain limited. To the best of our knowledge, the only pharmacokinetic data available are after a single oral dose in healthy Chinese individuals (Yang et al., 2017). Given the clinical demand to assess the potential of TET in the treatment of COVID-19 and its large V (Ju et al., 2015; Liu et al., 2023) that makes it difficult to evaluate the anti-SARS-CoV-2 activity of TET accurately based on plasma concentration, assessing TET concentration in the lungs is a crucial step. Currently, the physiologically based pharmacokinetic (PBPK) model is an important approach for predicting actual lung concentrations based on specific drug doses administered to the human body or for reverse engineering the dosing regimen based on the target concentration to be achieved (Sjögren et al., 2013; Mathew et al., 2021). Compared to traditional methods in which lung concentrations are directly predicted through blood drug concentrations or *in vitro* lung cell models, the PBPK model not only considers the dynamic equilibrium of drug concentrations between the lungs and blood plasma but also takes into account the distribution characteristics and mechanisms of drugs in the entire body. This makes the PBPK model more practical for predicting drug concentrations in the lungs and other tissues. To achieve this goal, a mechanistic animal PBPK model of TET was established based on the *in vitro* and *in vivo* drug metabolism and pharmacokinetic (DMPK) data of animals. Once fully verified, this model can be reasonably converted to a human PBPK model. The anticipated drug exposure in the lung can be determined once it has been validated through reported human pharmacokinetic profiles. Finally, the *in vivo* antiviral effects of TET were scientifically assessed using the relationship between the administered dose-exposure concentration at the lung target site and *in vitro* antiviral activity established through the PBPK model.

## 2 Materials and methods

### 2.1 Materials

Tetrandrine (purity >99.2%) was obtained from Zhejiang Conba Pharmaceutical Co., Ltd. Buspirone hydrochloride and dimethylsulfoxide (DMSO) were purchased from Sigma-Aldrich. High-performance liquid chromatography (HPLC)-grade acetonitrile and methanol were procured from Thermo Fisher, while HPLC-grade formic acid was obtained from Dikma. Purified water was sourced from Hangzhou Wahaha Group Co., Ltd.

### 2.2 Drug-specific parameters

#### 2.2.1 *In Vivo* preclinical PK studies

Male Sprague–Dawley (SD) rats (200–220 g) and male beagles (9–11 kg) were procured from Beijing Vital River Laboratory Animal Technology Co., Ltd. (Beijing, China) and SPF Biotechnological Co., Ltd. (Beijing, China), respectively. All animal experiments were reviewed and approved by the Institutional Animal Care and Use Committee of the research center and were in accordance with the animal health and welfare guidelines of the Association for Assessment and Accreditation of Laboratory Animal Care International (IACUC-DWZX-2021–662). Intravenous pharmacokinetic (PK) studies were conducted in the rats and dogs to refine and optimize the clearance and distribution processes.

The PK studies in the male SD rats were conducted following administration of an intravenous bolus dose of TET at 5 mg/kg and oral administration of TET at 15, 30, and 45 mg/kg. Blood samples were collected from the subjects 0.033 to 144 h after administration, with three animals being used for each dose level. In the PK studies in the male beagles, TET was administered via single intravenous bolus at a dose of 5 mg/kg and oral administration at doses of 10 and 15 mg/kg. Blood samples were collected from the subjects 0.033 to 144 h after administration, with data from five dogs being made available for each dose level.

#### 2.2.2 *In vitro* absorption, distribution, metabolism, and excretion (ADME) properties

##### 2.2.2.1 Physicochemical properties

The key physicochemical parameters of TET are shown in Table 1, and its solubility was determined. The dissociation constant (pKa) was then fitted using a series of measured solubility values of TET at different pH conditions (Table 2) and the built-in models in GastroPlus software.

**TABLE 1:** ADME parameters of TET used in the construction of the PBPK model.

ADME-related property	Parameter		Unit	Value	Method
Physicochemical	Molecular weight		g/mol	622.77	Calculated
	pKa		—	8.891	Fitted
	LogP		—	7.04	Literature
	Solubility (pH = 6)		mg/mL	1.93	Measured
Absorption	P_eff_	Rat	×10^−5^ cm/s	0.1692	IVIVE
		Dog		2.1071	
		Human		0.7616	
	f_u,ent_	Rat	%	10	Fitted
		Dog		1	
		Human		10	
	First pass effect (FPE, intestinal)	Rat	%	65	Fitted
		Dog		65	
		Human		65	
Distribution	f_u,p_	Rat	%	1.68	Measured
		Dog		0.68	
		Human		0.77	
	Adjusted f_u,p_	Rat	%	1.1584	Adjusted^a^
		Dog		0.5794	
		Human		0.3009	
	R_b/p_	Rat	—	3.08	Measured
		Dog		5.46	
		Human		3.32	
Metabolism	CL_h_	Rat	L/h	0.325	IVIVE
		Dog		7.837	
		Human		12.814	

^a^ The plasma protein binding (PPB) value obtained by the rapid equilibrium dialysis method assumes 100% binding to plasma albumin; the real PPB is adjusted based on the octanol-to-water-partition coefficient (LogP) considering lipid-related binding.

**TABLE 2:** Solubility of TET in phosphate-buffered saline solution at different pH values.

pH	Solubility (mg/mL)
7.4	0.000325
6.5	0.492
6.0	1.93
5.5	9.86
5.0	11.5
4.0	15
2.5	88.6

##### 2.2.2.2 Solubility determination at different pH values

To ascertain the solubility at different pH values, a series of phosphate-buffered saline (PBS) solutions spanning a pH range of 2.5–7.4 was prepared according to the formula outlined in the pharmacopoeia. Excess solid TET was introduced to the PBS solutions at different pH levels until no further dissolution occurred. The mixture was then vortexed and sonicated for 10 min to facilitate dissolution, followed by shaking at 200 rpm on an oscillator at room temperature for 24 h. The vortexing and sonication were repeated 3–4 times during this period to aid dissolution. Subsequently, the samples were centrifuged at 18,800*g* for 10 min, and the resulting supernatant was filtered through a 0.45 µm membrane. The obtained solutions were further processed as described and analyzed using liquid chromatography with tandem mass spectrometry (LC-MS/MS).

##### 2.2.2.3 *In vitro* metabolic stability

The well-stirred model (Rowland et al., 1973; Pang and Rowland, 1977) was used to obtain the hepatic clearance (CL_h_) from the intrinsic clearance (CL_int_), hepatic blood flow (Q_h_), blood-to-plasma partition (R_b/p_), and unbound fractions in plasma (f_u,p_) and liver microsomes (f_u,mic_) as follows:

CLint=0.693in vitro t12×volume of incubation μLamount of microsomal protein in incubation mg×45mg microsomesg liver×g liverkg body weight

$${CL}_{h}=\frac{Q_{h}\times f_{u,p/}R_{b/p}\times {CL}_{\mathrm{int}}\;/\;f_{u,mic}}{Q_{h}+{CL}_{\mathrm{int}}\cdot f_{u,p}{/R}_{b/p}\;/\;f_{u,mic}}$$

where the hepatic blood flow (Q_h_) values for rats, dogs, and humans were taken as 55.2, 30.8, and 20.7 mL/min/kg, respectively (Davies and Morris, 1993); the f_u,p_, f_u,mic_, and R_b/p_ values were measured in our previous work (Liu et al., 2023).

##### 2.2.2.4 Permeability of TET in Caco-2 monolayers

The apparent permeability coefficient (P_app_) was obtained from the Caco-2 cell transcellular transport assay. Caco-2 cell lines were cultured (with passage number of 20, ATCC, Manassas, VA, United States) as described previously (Gao et al., 2020). The cells were cultured for 20–22 days after seeding and then evaluated by measuring the transepithelial electrical resistance (TEER) (Millicell ERS^®^, Millipore Corporation) before transport experiments. The monolayers were considered tight enough for the transport experiments once the TEER values exceeded 1,200 Ω cm^2^. Transepithelial permeability studies were then conducted for TET (2 μM) and the control compounds (atenolol, 2 μM, low permeability; metoprolol, 2 μM, high permeability; digoxin, 2 μM, P-gp substrate) from the apical to basolateral (AB) and basolateral to apical (BA) sides for 120 min. At 60, 90, and 120 min, about 50 µL of the solution was collected from the receiver side, and the same volume of Hanks’ balanced salt solution was immediately added to maintain the volume. After completion, 50 µL aliquots were collected from both the apical and basolateral compartments. The collected samples were processed and analyzed by LC-MS/MS. The P_app_ and efflux ratio (ER) values were calculated using the following equations:

$$P_{\text{app}}=\frac{1}{A\times C_{0}}\times \frac{d_{x}}{d_{t}}$$

$$\text{ER}=\frac{P_{\text{app},\text{BA}}}{P_{\text{app},\text{AB}}}$$

where d_x_/d_*t*_ is the slope of the linear plot of the cumulative amount of drug transported versus time, A is the surface area of the filter insert (cm^2^), and C_0_ is the initial concentration of the compound at the dosing side.

#### 2.2.3 Bioanalytical method

The concentrations of TET and the control compounds were determined in various biomatrices using LC-MS/MS, as reported previously (Wang et al., 2022). Briefly, all samples were precipitated with acetonitrile to remove the proteins. Analytes of compounds of interest were separated using a C18 column (3.0 mm × 50 mm, 2.6 μm, Phenomenex). Multiple reaction monitoring (MRM) was selected for detection in the positive ion mode at *m*/*z* 609.0→381.1 for TET and *m/z* 386.4→122.1 for internal standard, buspirone.

#### 2.2.4 Data analysis

The experimental data are shown as mean ± standard deviation (SD). The PK parameters including clearance (CL), distribution volume (V), AUC_(0-*t*)_, AUC_(0-∞)_, and mean response time (MRT) were calculated using WinNonlin 7.0 (Pharsight, CA) through the non-compartmental analysis model.

### 2.3 PBPK modeling strategy

#### 2.3.1 Model structure and simulation

**FIGURE 2:**
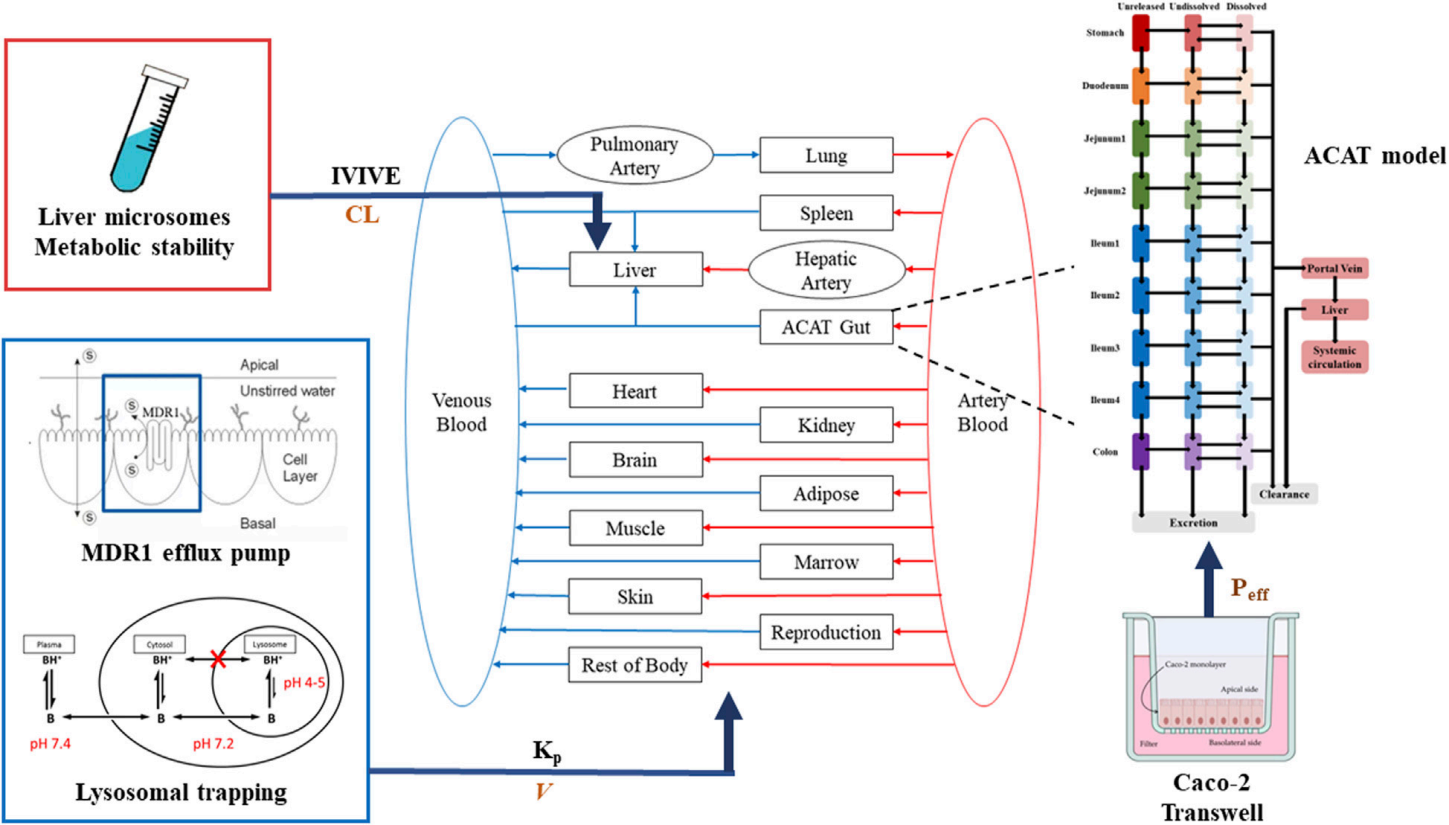
Schematic of the whole-body PBPK model depicting the disposition of TET.

The PBPK model for TET was constructed in GastroPlus platform (versions 9.8) (Simulations Plus, Inc., United States). A middle-out approach was used to develop the PBPK model by integrating available *in vitro* or *in vivo* data. The whole-body PBPK model included the gastrointestinal tract, spleen, liver, kidneys, lungs, heart, muscles, adipose tissues, reproductive organs, and rest of the body. Each tissue was defined as a single ‘‘well-stirred’’ compartment, where the drug molecules entered and exited tissues through the blood flow in an instantaneous equilibrium perfusion manner (Figure 2).

The oral absorption was predicted using the advanced compartmental absorption and transit (ACAT) model, which is a semiphysiological transit model consisting of nine compartments corresponding to different segments of the gastrointestinal tract, as implemented in GastroPlus. The fasted Opt logD models of rat, dog, and human were used to simulate oral absorption in all species tested. The P_app_ values of TET and positive control drugs (atenolol and metoprolol) obtained from the Caco-2 monolayer cell model were used to predict the effective permeability across the enterocyte (P_eff_) by combining with the implemented *in vitro*/*in vivo* correlation model. In addition, the unbound fraction of enterocytes (f_u,ent_) was optimized by considering the involvement of lysosomal trapping to achieve the observed *t*_max_ and C_max_.

Based on the extensive metabolic features of TET and identification from a previous mass balance study for minor recovery of the parent drug in the feces and urine of rat (much less than 1%), hepatic metabolism was defined as the overall clearance mechanism. Thus, the *in vitro* clearance determined for the liver microsomes was converted to *in vivo* clearance using the in-vitro-to-in-vivo extrapolation (IVIVE) method. The TET-related tuning of ADME parameters for the development of the PBPK model are summarized in Table 1.

Generally, the overall tissue distribution of a compound is reflected in the distribution volume at steady state (V_ss_), which is mainly controlled by the protein binding rate, R_b/p_, LogP, pKa, etc. However, we reported in our previous work that the cellular exposure of TET is associated with lysosomal trapping and P-gp-mediated efflux (Wang et al., 2022). It is difficult to extrapolate the V_ss_ of TET using the IVIVE approach owing to the varying lysosome content and P-gp expressions in different cells of different organs. In this case, the overall V_ss_ of TET was defined in two ways. The V_ss_ of liver, spleen, lungs, brain, adipose tissues, and muscles were defined using the K_p_ values measured from the rat tissue distribution experiment in the model (Liu et al., 2023). The V_ss_ values of the other tissues were defined through calculation of the K_p_ values according to the Lukacova–Rodgers single method. The K_p_ values of different tissues for construction of the PBPK model are shown in Table 3.

**TABLE 3:** Tissue partition coefficients (K_p_) of the TET inputs for construction of the rat PBPK model that is further extrapolated to dog and human PBPK modeling.

Tissues	K_p_	Obtained method
Spleen	802	Measured
Liver	603	Measured
Lung	458	Measured
Heart	149.69	Predicted
Kidney	143.45	Predicted
Reproductive organ	69.29	Predicted
Yellow marrow	49	Predicted
Muscle	32.3	Measured
Brain	25.7	Measured
Red marrow	23.22	Predicted
Skin	10	Predicted
Rest of the body	10	Predicted
Adipose	8.68	Measured

#### 2.3.2 Interspecies scaling and PBPK simulations for animals and humans

PBPK models of the different preclinical species were fully validated before the human PBPK simulations. For translation to dogs and humans, the model was adapted to include dog and human organ volumes, cardiac output, hematocrit, and relative blood flow to each organ. TET clearance was obtained by the IVIVE method based on the measured *in vitro* clearance of liver microsomes in beagles and humans. The distribution volumes were corrected from the generated V values of rats based on the plasma protein binding (PPB) of beagles and humans. Intestinal absorption values were obtained using the *in vitro* membrane permeability coefficients from the Caco-2 monolayer cells converted from different species.

#### 2.3.3 Parameter sensitivity analysis

Given the potential impacts of lysosomal trapping on the absorption of TET in the intestine, a parameter sensitivity analysis was specifically carried out to elucidate the effects of the unbound fractions of TET in the enterocytes (f_u,ent_) on *t*_max_ and C_max_. The sensitivity analysis helps in understanding how the changes to a particular parameter affect the overall outcomes or responses of a system or model. In this case, the focus was on assessing the influences of f_u,ent_ on the PK properties of TET, particularly *t*_max_ and C_max_.

#### 2.3.4 Model validation

Simulation of dog PK via i.v. and oral administrations to verify the model can adequately predict PK behaviors through the scaling up method. The PK profiles of Chinese subjects reported in literature post single oral administration of TET (100 mg) were utilized to further verify the PBPK model according to the physiological characteristics of Chinese people (Yang et al., 2017). The accuracy of this PBPK model for predicting PK features was quantitively evaluated through the AUC and C_max_. The goodness of prediction was assessed using the fold error (FE) by calculating the ratio of predicted to observed values, with FEs of 0.5–2 being considered as acceptable.

#### 2.3.5 Model application

The verified TET PBPK model was finally applied to predict the unbound concentration in the lungs when the dosing regimen of TET was set to the recommended dosage and duration in the drug leaflet for silicosis. The TET simulation was designed based on administration of 100 mg thrice a day (t.i.d.) for 6 days. The therapeutic effects of TET against SARS-CoV-2 were explored by comparing the *in vitro* EC_90_ value (1.52 μg/mL, Liu et al. (2023) with the target exposure level in the lungs.

## 3 Results

### 3.1 PK behaviors of TET in rats and dogs

The mean plasma concentration versus time profiles in rats and dogs after various i.v. (A) and p.o. (B) doses of TET as well as the corresponding PK parameters are presented in Tables 4, 5. After an i.v. bolus of TET was administered, similar PK features of low clearance and large distribution volume were observed in both rats and dogs. After p.o. administration of TET, the systemic exposures (in terms of AUC) in rats and dogs increased in a dose-dependent manner. The absorption of TET was relatively slow based on the time to reach C_max_ (*t*_max_ within 6–36 h). The elimination duration of TET was much larger in rats and dogs. The average bioavailability of TET was moderate, with a slightly higher bioavailability being observed in dogs.

**TABLE 4:** Corresponding pharmacokinetic parameters of rats after various intravenous (i.v.) and oral (p.o.) doses of TET (n = 3).

Species	Parameter	Unit	*i.v*	*p.o*		
			5 mg/kg	15 mg/kg	30 mg/kg	45 mg/kg
Rat	*t*_1/2_	h	21.1 ± 1.36	52.3 ± 6.84	45.9 ± 23.4	40.1 ± 6.97
	*t*_max_	h	—	12.0 ± 10.5	6.67 ± 4.62	36.0 ± 52.0
	C_0_	ng/mL	525 ± 126	—	—	—
	C_max_	ng/mL	—	101 ± 46.2	169 ± 61.1	265 ± 53.9
	AUC_(0-*t*)_	h·ng/mL	5,942 ± 537	3,534 ± 1,358	8,992 ± 2,602	13,117 ± 5,856
	AUC_(0-∞)_	h·ng/mL	6,166 ± 573	4,091 ± 1,745	12,444 ± 6,681	13,902 ± 6,490
	MRT_(0-*t*)_	h	33.7 ± 1.42	57.3 ± 7.62	84.4 ± 41.8	56.2 ± 11.2
	V	L/kg	24.8 ± 0.952	—	—	—
	CL	mL/h/kg	815 ± 72.1	—	—	—
	F	%	—	22.1	33.6	25.1

**TABLE 5:** Corresponding pharmacokinetic parameters of dogs after various i.v. and p.o. doses of TET (n = 5).

Species	Parameter	Unit	*i.v*	*p.o*	
			5 mg/kg	10 mg/kg	15 mg/kg
Dog	*t*_1/2_	h	39.2 ± 9.82	74.4 ± 19.9	60.0 ± 15.0
	*t*_max_	h	—	25.6 ± 39.4	13.6 ± 6.07
	C_0_	ng/mL	692 ± 153	—	—
	C_max_	ng/mL	—	50.9 ± 17.9	70.9 ± 63.7
	AUC_(0-*t*)_	h·ng/mL	4,098 ± 474	2,536 ± 773	5,255 ± 4,683
	AUC_(0-∞)_	h·ng/mL	4,495 ± 506	3,639 ± 1,377	6,421 ± 6,040
	MRT_(0-*t*)_	h	52.7 ± 12.8	106 ± 34.8	91.8 ± 23.1
	V	L/kg	63.0 ± 13.8	—	—
	CL	mL/h/kg	1,124 ± 132	—	—
	F	%	—	40.5	47.6

### 3.2 ADME profiles of TET

**FIGURE 3:**
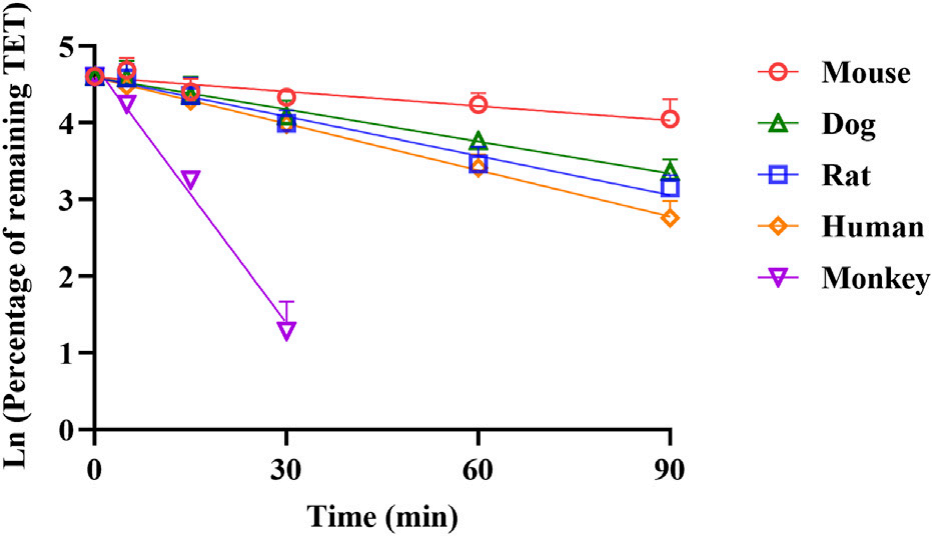
Comparison of NADPH-depended depletion profiles of TET in the liver microsomes of different species (n = 3).

Robust information on the ADME-related properties of a compound is linked to a comprehensive understanding of its DMPK behaviors, which facilitates the construction of a mechanistic PBPK model based on the bottom-up approach. The obtained ADME properties of TET are summarized in Table 1. Aside from the values of pKa and f_u,ent_ that were derived from fitting method, the other values were generated from *in vitro* models. The physicochemical properties, distribution, and metabolism-related data were utilized to build the i.v. PBPK model. By integrating the absorption-related data, the oral PBPK model was constructed finally. The apparent permeability of TET across the Caco-2 cell monolayers from the AB side with a P_app_ of 2.38 ± 0.47×10^−6^ cm/s was transferred to P_eff_ for transport across the small intestinal segments based on the built-in algorithm. The resulting P_eff_ values for rats, dogs, and humans ranged from 1.69 × 10^−6^ to 21.07 × 10^−6^ cm/s, implying different intestinal absorption in different species. The *in vitro* metabolic stability curves of TET derived from the liver microsomes of various species are presented in Figure 3. The resulting parameters, namely *t*_1/2_ and corresponding CL_int_ in rat, dog, and human liver microsomes were 40.7 ± 3.1, 49.9 ± 1.6, and 34.7 ± 4.0 min as well as 61.5 ± 4.5, 40.0 ± 1.3, and 46.7 ± 5.3 mL/min/mg protein, respectively. Here, the *t*_1/2_ values were used to convert the *in vivo* clearances owing to the well-fitted outcomes in rat and dog i.v. PBPK modeling and simulation.

### 3.3 Construction of the TET PBPK model in rats

**FIGURE 4:**
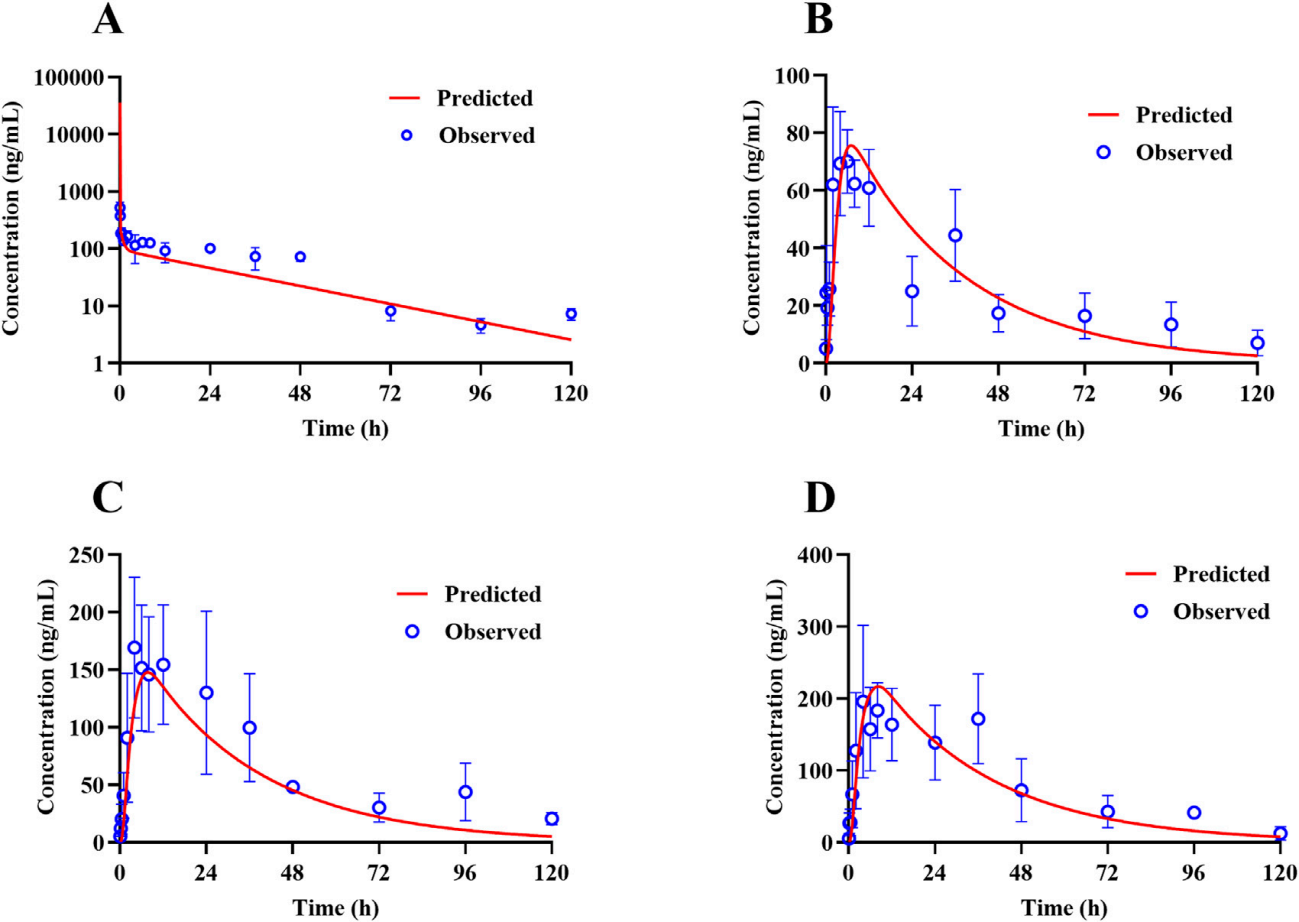
Representative observed (symbols) and PBPK model fitted (lines) pharmacokinetic profiles of TET in the plasma of rats following **(A)** a single i.v. bolus administration of 5 mg/kg of TET and **(B–D)** single oral administration of 15–45 mg/kg of TET .

**FIGURE 5:**
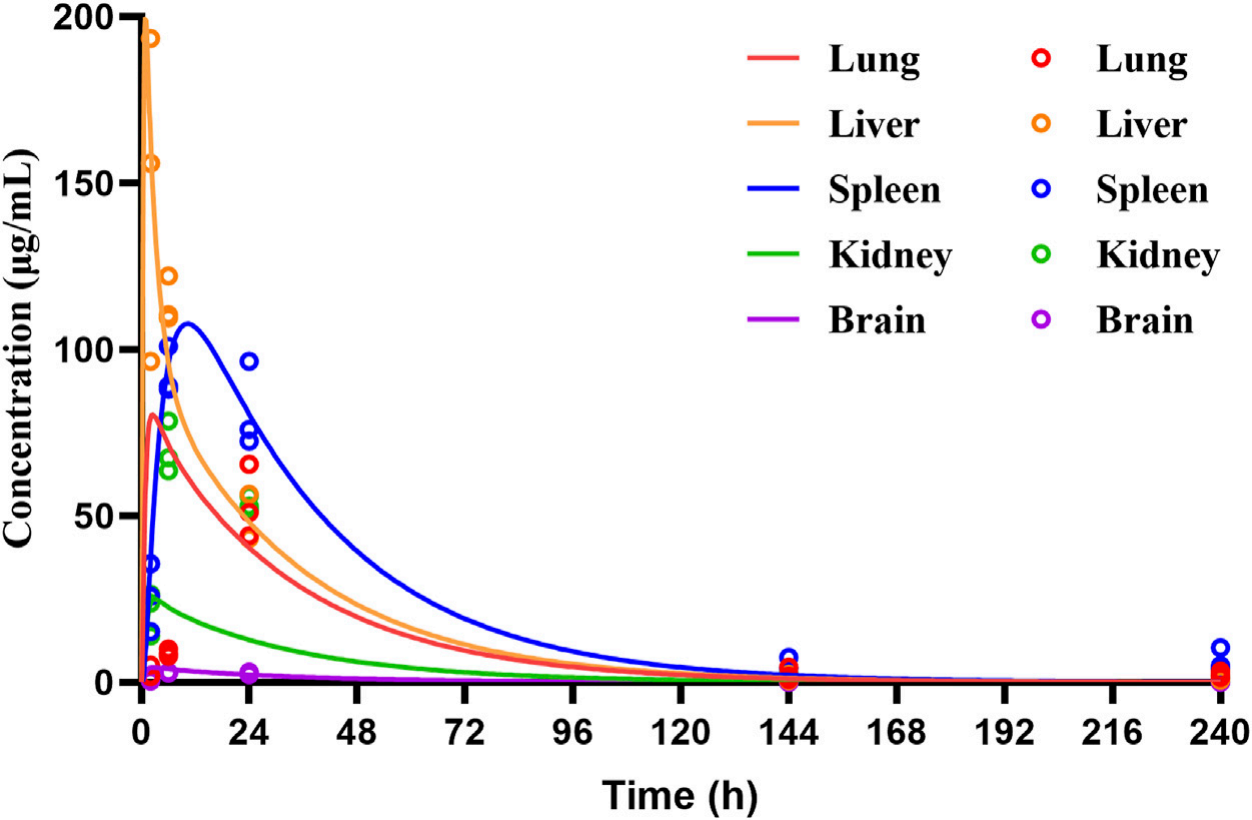
Temporal concentrations of TET in different tissues of rats following single oral administration of 30 mg/kg of TET. The symbols represent the observed values, while the lines represent the PBPK model predicted profiles.

The PBPK model for rats via i.v. route was initially built using the TET-related physicochemical parameters, *in vitro* hepatic clearance, and distribution volume generated from the determined K_p_ values of major tissues along with the fitted K_p_ values of other organs. The simulated plasma concentration versus time profile was able to capture the observed concentrations over different time points after i.v. administration in rats at 5 mg/kg (Figure 4A). The predicted plasma concentrations described by the AUC_(0-∞)_ were all close to the observed values (Table 6). Thus, the assumptions of well-stirred tissue compartments and hepatic clearance are reasonable for the basic TET PBPK model. Next, the oral PBPK model of TET in rats was further optimized on the basis of the i.v. PBPK model by combining the ACAT model with P_eff_ and f_u,ent_. The P_eff_ value was directly converted from P_app_ obtained from the Caco-2 cells, while the f_u,ent_ value was fitted since it was proved that lysosomal trapping is involved in the intracellular distribution of TET. The simulated plasma concentration versus time profiles for oral doses of 15–45 mg/kg agreed well with the observed concentrations over different time points (Figures 4B–D). Additionally, reasonable predictions of TET concentrations were obtained for the major tissues, including lungs, spleen, liver, and kidneys (Figure 5). The predicted PK parameters were less than twice the observed values (Table 6).

**TABLE 6:** Prediction performances of the PBPK model for rats and dogs.

Species	Dose	Source	C_max_	AUC_(0-*t*)_	AUC_(0-∞)_
			(ng/mL)	(ng·h/mL)	(ng·h/mL)
Rat	15 mg/kg	PBPK	75.6	2,836	2,922
		Observed	70.1	3,027	3,284
		Ratio	1.08	0.94	0.89
	30 mg/kg	PBPK	147.7	5,638	5,811
		Observed	169.3	8,146	8,817
		Ratio	0.87	0.69	0.66
	45 mg/kg	PBPK	216.9	8,423	8,682
		Observed	195.7	10,030	10,280
		Ratio	1.11	0.84	0.84
Dog	10 mg/kg	PBPK	56.7	3,375	3,655
		Observed	44	2,707	3,136
		Ratio	1.29	1.25	1.17
	15 mg/kg	PBPK	84.8	5,208	5,479
		Observed	70.4	5,506	6,319
		Ratio	1.2	0.95	0.87

### 3.4 Validation of the TET PBPK model in dogs

**FIGURE 6:**
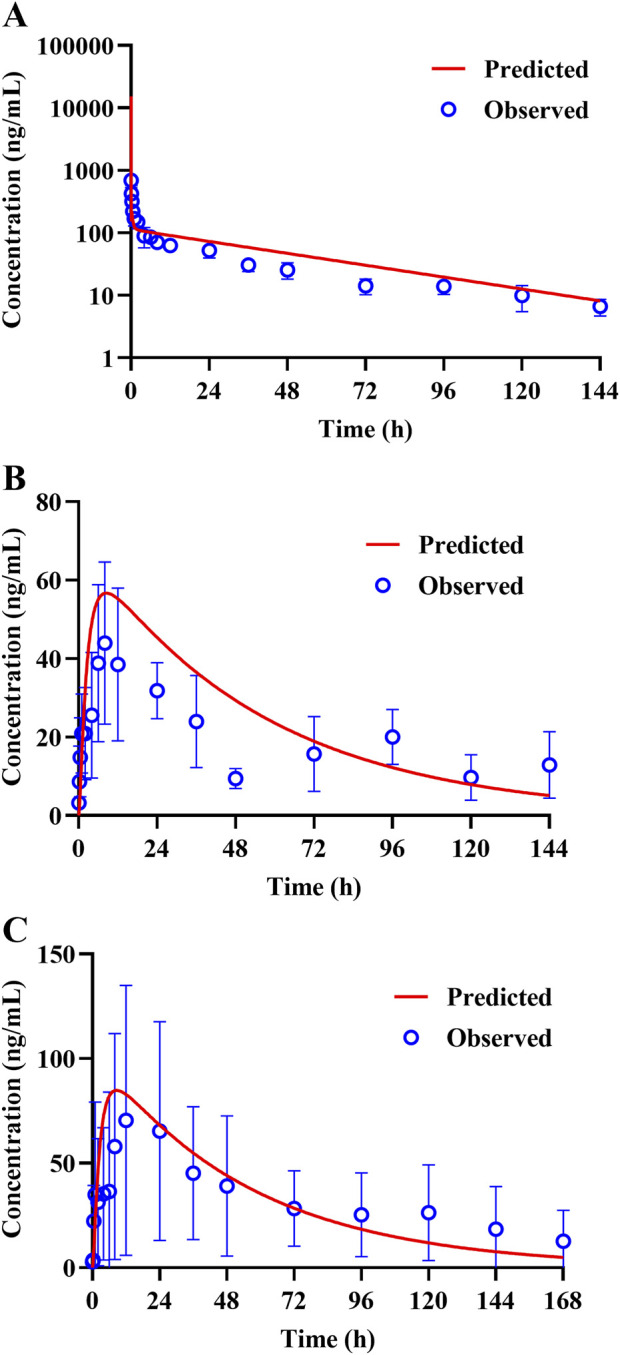
Representative observed (symbols) and PBPK model fitted (lines) pharmacokinetic profiles of TET in the plasma of dogs following **(A)** a single i.v. bolus administration of 5 mg/kg of TET and **(B, C)** single oral administrations of 10 and 15 mg/kg of TET.

The constructed rat PBPK model was validated through translation to dogs based on the same simulation framework and the physiological parameters of dogs. The plasma concentration data of TET after i.v. administration were described well in the dog PBPK model (Figure 6A), which indicated that the IVIVE-based hepatic clearance and *in-vivo*-informed K_p_ values could capture the clearance and distribution processes of TET. Regarding the oral PBPK model for dogs, aside from f_u,ent_ that was fitted according to the observed C_max_ and *t*_max_, the P_eff_ values for dogs derived from P_app_ incorporated the dog ACTA model. The predicted PK profiles agreed well with the observed PK profiles for doses ranging from 10 to 15 mg/kg (Figures 6B, C). The corresponding PK parameters are summarized in Table 6. Overall, the EFs ranged between 0.87 and 1.29.

### 3.5 Simulated exposure relative to reported anti-SARS-CoV-2 activity in lungs based on the human PBPK model

**FIGURE 7:**
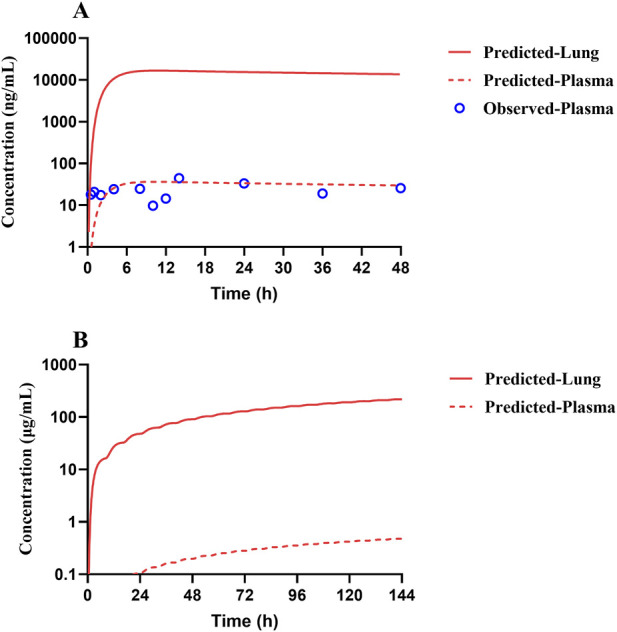
Predicted (lines) and observed (symbols) TET exposure profiles from the plasma and lung of a typical Chinese after **(A)** a single oral dose of 100 mg and **(B)** repeated oral doses of 100 mg per session thrice a day for 6 consecutive days according to the recommended dosing regimen for treating silicosis. The clinical data were obtained from literature.

Human PK projection for TET from animal pharmacokinetics was ultimately performed using the validated PBPK model. The plasma concentration versus time profiles of healthy Chinese male volunteers after oral administration of 100 mg of TET were obtained from literature and found to be described well by the human PBPK model (Figure 7A). Owing to the lengthy half-life of TET, capturing a complete PK curve requires lengthy sample collection times. However, the available literature only reported data within 48 h; thus, the predicted results mainly testified that the absorption phase was in good agreement. Based on the lung K_p_ values obtained from animal experiments, the PBPK model projected that the pulmonary concentration of TET in the human body reached 16.7 μg/mL (free unbound concentration of 0.13 μg/mL) under the same dose regimen (single oral administration at 100 mg), which is unable to achieve therapeutic effects against SARS-CoV-2 (1.52 μg/mL). Finally, it was predicted that the lung concentration could reach 218 μg/mL (free unbound concentration of 1.74 μg/mL), which exceeds the *in vitro* efficacy concentration according to the medication regimen for treating silicosis (100 mg thrice daily for 6 consecutive days) (Figure 7B).

## 4 Discussion

Drug repurposing is a powerful solution for emerging diseases, such as COVID-19, as repurposing-approved drugs can be offered as medication to patients faster and at less cost than through the *de novo* drug discovery process (Parvathaneni et al., 2019; Arshad et al., 2020; Zhou et al., 2020; Roessler et al., 2021). However, there is a huge gap between *in vitro* antiviral activity and *in vivo* efficacy. Systematic analyses in the context of plasma and target-site exposures that are achievable after administration of the approved doses to humans are highly important in drug repurposing. Although TET has been clinically used for many years, existing knowledge shows that there is a lack of in-depth PK investigations, especially PK data from humans. The PK properties of a drug determine its ability to reach the target site and exert the intended pharmacological effects. Considering that SARS-CoV-2 mainly attacks the lungs, the potential of a therapeutic agent to accumulate in lung tissues rather than in plasma may provide direct rational evidence (Mouton et al., 2008; Zhang et al., 2019; Zang et al., 2022). However, the absence of observed exposure data for almost all licensed drugs in human lungs following approved dosing is a major obstacle in the determination of their potential antiviral efficacies. Simulated concentrations in the lungs derived from the PBPK model are well accepted in literature (Rodgers and Rowland, 2007; Yeo et al., 2020; Jeong et al., 2022). Therefore, in the current investigation, a validated PBPK framework was developed to harvest the anticipated concentration versus time profiles of TET in human lungs based on integrated comprehensive animal whole-body disposition studies and available human PK profiles.

In view of the study objectives and available data, the workflow in the present investigation included the following steps: First, in-house PK studies were conducted after i.v. and oral dosing in rats and beagles, followed by biodistribution studies in rats and *in vitro* experiments to generate TET-specific ADME characteristics of different species. Second, a rat i.v. PBPK model was initially established, which was then transferred to beagles for validation. The ACTA model and intestinal absorption parameters were introduced to build the oral PBPK model for rats, which was further validated with the oral PK results of beagles. Third, the validated oral PBPK model was translated to humans based on published human PK data (Yang et al., 2017). Lastly, the full concentration versus time profiles of TET in the plasma and lung tissues of humans after oral administration were predicted according to the clinically recommended application dose and regimen.

It is obvious that a major challenge needs to be addressed in the current approach with the aid of PBPK modeling, namely rational bridging of the species-barrier gap during the drug disposition and tissue distribution processes. The physicochemical properties of a compound, such as the molecular weight, LogP, solubility, and pKa, are closely related to its *in vivo* disposition processes, including absorption, distribution, and elimination. Therefore, the PBPK model can predict results closer to real conditions in the body only through comprehensive and accurate physicochemical property parameters of TET (Tsamandouras et al., 2015; Zhuang and Lu, 2016). The initial PK outcomes of rats and dogs display similar characteristics, with low systemic clearance and high distribution volume, which are in agreement with the results of rat tissue distribution experiments. The i.v. PBPK model based on the two main parameters of clearance and distribution volume is the basis for PBPK modeling. In building the basic i.v. PBPK model of TET in rats, the *in vivo* clearance was easily fitted by applying the IVIVE approach based on the CL_int_ values obtained from *in vitro* NADPH-dependent degeneration, indicating that TET is mainly eliminated via hepatic metabolism. While fitting the *in vivo* V values, various algorithms were attempted, which could not capture the observed V trends well. Finally, the Lukacova–Rodgers algorithm (Sherbetjian et al., 2022) was applied to calculate the total V in combination with the major tissue K_p_ values determined from rat tissue distribution experiments (Table 3), which produced results closer to the observed value (24.8 L/kg). Our previous investigations showed that the tissue distribution features of TET are influenced by a few factors, such as lysosomal trapping; further, TET is a substrate for P-gp but not for OATPs or OCTs (Wang et al., 2022). The predicted K_p_ and corresponding V values calculated using the algorithm by considering the roles of lysosomes are much higher than the measured values. Thus, we included lysosome-enriched tissues (liver, lungs, and spleen) and the brain that have pronounced P-gp efflux, as well as muscle and adipose tissues with larger masses, as measured values of rat tissue distribution; the K_p_ values for the other tissues were calculated using the Lukacova–Rodgers algorithm and aligned with the measured values of solubility, pKa, f_u_, and R_b/p_. It was found that the V values fitted in this manner were very close to the *in vivo* data. The i.v. PBPK model developed using the middle-out approach thus offers an important foundation for the oral model.

The oral PBPK model is relatively complex owing to the addition of the intestinal physiological ACTA model and absorption–metabolism mechanisms of TET. For the absorption rate, we applied the *in vitro* P_app_ measured from the Caco-2 cells converted to *in vivo* P_eff_ for characterization. It is noted that the same P_app_ value corresponds to large differences in P_eff_ for rats, beagles, and humans, with the *in vivo* absorption rate in beagles being 12 times higher than that in rats and 3 times higher than that in humans (Table 1); this suggests that the intestinal absorption capacity in beagles is much higher than in other species. When the P_eff_ value was incorporated in the ACTA model, it was observed that C_max_ could be predicted accurately, but it was unable to capture *t*_max_. Considering the lysosomal capture characteristics of TET and the observed recovery rate of the AB side transport in Caco-2 cell experiments being merely 41%, it is possible that lysosomal capture significantly influences the absorption rate of TET during its passage through the intestinal epithelial cells. The parameter sensitivity analysis revealed a strong correlation between f_u,ent_ (% of unbound enterocytes) and *t*_max_ (Supplementary Figure S1). By setting f_u,ent_ to 10% for rats, excellent agreement was achieved with the measured *t*_max_ (Table 6). However, it was found that both *t*_max_ and C_max_ were much higher than the measured values in the oral model of dogs when defined with the same parameters; these values matched the measured *t*_max_ and C_max_ only upon reducing f_u, ent_ to 1% (Table 6). In the human oral PBPK model, the results for 10% and 1% f_u, ent_ were compared, and it was found that the predicted *t*_max_ and C_max_ values at f_u, ent_ of 10% were closer to the measured values reported in literature. Hence, it is suggested that the intestinal absorption profile of TET in humans may be more like that of rats and differs greatly from that of beagles.

Since the high tissue distribution characteristics of TET can lead to an underestimated *in vivo* exposure–response relationship solely based on plasma concentration (Zhang et al., 2019), the significance of PBPK modeling is highlighted. Thus, adequate preclinical DMPK studies combined with sophisticated data analyses are the best approaches for developing mechanistic models elucidating drug exposure in human local tissues, especially the lungs that are the target tissues of coronavirus infections, and directly correlating drug efficacy. The blood-concentration-validated human oral PBPK model after single dosing yielded the time–concentration profile of TET in the human lungs, where the peak concentration of 16.7 μg/mL (free concentration of 0.13 μg/mL) failed to reach the previously suggested *in vitro* anti-SARS-CoV-2 concentration (1.52 μg/mL) (Liu et al., 2023). The drug concentration versus time curves of TET in both plasma and lung tissues were ultimately predicted according to the dosing regimen recommended for the treatment of silicosis (100 mg per oral dose thrice daily for 6 days). The results suggest that the free concentration of TET in the lungs increased gradually and reached 1.52 μg/mL at 110 h (∼4.5 days) after administration that was maintained thereafter until the end of treatment. The lung exposure outcome predicted by the PBPK model offers confidence in the use of TET to treat SARS-CoV-2 infection.

The current investigation provides insights into drug partitioning in the lungs that are not accessible directly and/or are challenging to sample in humans, serving as an alternative method for evaluating antiviral activity. However, there are some obvious limitations of this work. Despite predictions from animal experiments and PBPK models, the complex structure of the lung introduces concentration variations in the alveolar epithelial cell surfaces, intracellular drug distribution, and upper and lower airways that ultimately impact the drug efficacy (Bäckman et al., 2018; Alsmadi et al., 2023). Furthermore, changes in the physiological environment of the lungs during respiratory viral infections and disease states can influence drug distribution and exposure (Chityala et al., 2020; Wang et al., 2022). In our previous work, we observed that pulmonary exposure to TET in mice with lipopolysaccharide-induced pneumonia was more than twice of that observed in healthy mice (Wang et al., 2022). Although the current study and proposed model do not yet account for the impacts of disease states, the findings remain fundamentally accurate. In addition, when using the IVIVE method to predict interspecies clearance, although the predicted results for rats and dogs are consistent with real-world clearance observations, the predicted clearance of the model cannot be verified in humans owing to the lack of corresponding clearance rate data. Further validation will therefore be conducted once sufficient clinical PK data become available in the future.

In summary, through extensive non-clinical DMPK investigations, we developed a PBPK model that was specifically designed for oral administration of TET in humans to overcome interspecies differences. This model facilitated the determination of pulmonary drug exposure concentrations achieved according to the clinical dosing of TET. By comparing the *in vivo* target concentrations with *in vitro* efficacy concentrations, we rigorously evaluated the potential effectiveness of TET against COVID-19. The PBPK model of TET is expected to help with directly predicting the efficacy or toxicity of the drug concentration in the target organ, thereby enabling researchers to better adjust the dosage and administration of TET as well as providing a reference for precision medicine in clinical practice.

## Data Availability

The original contributions presented in the study are included in the article/Supplementary Material, and any further inquiries may be directed to the corresponding author.

## Supplementary material

SUPPLEMENTARY FIGURE S1 Parameter sensitivity analyses for the relationships between the unbound fractions of TET in the enterocytes (f_u,ent_) on *t*_max_ and C_max_.
