# Supplementary material for: Quantitative pulmonary pharmacokinetics of tetrandrine for SARS-CoV-2 repurposing: a physiologically based pharmacokinetic modeling approach
Source: Front Pharmacol. 2024 Sep 13;15:1457983. doi: 10.3389/fphar.2024.1457983 (PMC11427368; doi:10.3389/fphar.2024.1457983)
Supplement: Supplementary file 1 [file Image1.pdf]

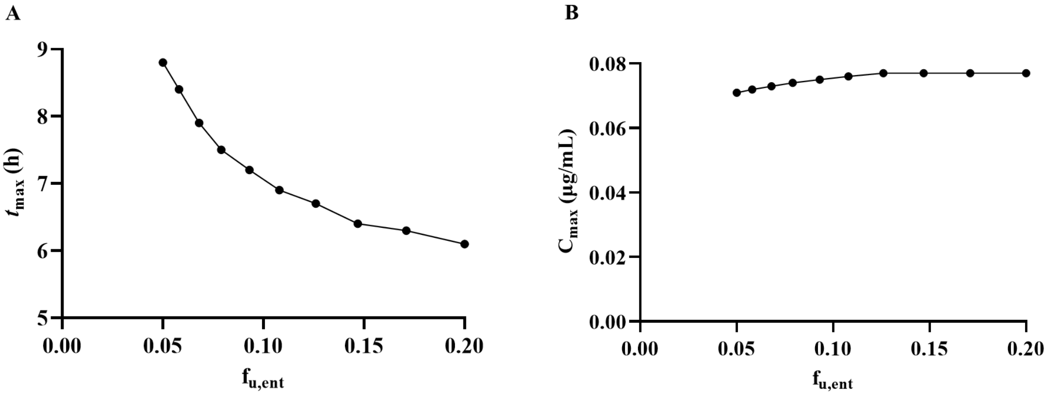

Fig. S1 The parameter sensitivity analysis of the unbound fraction in the enterocytes ($f_{u,ent}$) on t_{max} and C_{max} of TET.
